# Bioleaching and Electrochemical Behavior of Chalcopyrite by a Mixed Culture at Low Temperature

**DOI:** 10.3389/fmicb.2021.663757

**Published:** 2021-05-10

**Authors:** Tangjian Peng, Wanqing Liao, Jingshu Wang, Jie Miao, Yuping Peng, Guohua Gu, Xueling Wu, Guanzhou Qiu, Weimin Zeng

**Affiliations:** ^1^School of Minerals Processing and Bioengineering, Central South University, Changsha, China; ^2^Key Laboratory of Biometallurgy, Ministry of Education, Changsha, China; ^3^State Key Laboratory of Microbial Technology, Shandong University, Qingdao, China; ^4^CSIRO Process Science and Engineering, Clayton, VIC, Australia

**Keywords:** chalcopyrite bioleaching, low temperature, pure culture, mixed culture, electrochemical behavior

## Abstract

Low-temperature biohydrometallurgy is implicated in metal recovery in alpine mining areas, but bioleaching using microbial consortia at temperatures <10°C was scarcely discussed. To this end, a mixed culture was used for chalcopyrite bioleaching at 6°C. The mixed culture resulted in a higher copper leaching rate than the pure culture of *Acidithiobacillus ferrivorans* strain YL15. High-throughput sequencing technology showed that *Acidithiobacillus* spp. and *Sulfobacillus* spp. were the mixed culture’s major lineages. Cyclic voltammograms, potentiodynamic polarization and electrochemical impedance spectroscopy unveiled that the mixed culture enhanced the dissolution reactions, decreased the corrosion potential and increased the corrosion current, and lowered the charge transfer resistance and passivation layer impedance of the chalcopyrite electrode compared with the pure culture. This study revealed the mechanisms via which the mixed culture promoted the chalcopyrite bioleaching.

## Introduction

Bioleaching as a low-cost biotechnology offers an alternative to traditional pyrometallurgical methods to extract valuable metals from sulfide minerals (Hedrich et al., 2016; Banerjee et al., 2017; Pathak et al., 2017; Hu et al., 2020; Srichandan et al., 2020). Studies on bioleaching have been extensively performed using mesophilic, moderately thermophilic and extremely thermophilic acidophiles (Gu et al., 2013; Acosta et al., 2014; Li et al., 2018; Ai et al., 2019). On the contrary, bioleaching at low temperatures was only discussed in limited documents. Microbial oxidation of sulfide minerals could occur at relatively low temperatures (Ahonen and Tuovinen, 1991, 1992; Elberling et al., 2000). It was observed that the microbially mediated dissolution of sulfide minerals could reach 30% of the maximal value at 21°C (Langdahl and Ingvorsen, 1997). *Acidithiobacillus ferrivorans* is a well-known cold-adapted acidophile that can use iron and sulfur as a sole energy source (Kupka et al., 2007, 2009; Hallberg et al., 2010). Studies using *At. ferrivorans* in bioleaching of sulfide minerals at low temperatures have been performed. The leaching rate of chalcopyrite at 4°C using *At. ferrivorans* was higher than using the mesophilic *At. ferrooxidans* (Dopson et al., 2007). It was found that S^0^ formed during chalcopyrite bioleaching and the content of S^0^ increased until dissolution ceased, suggesting S^0^ passivation (Liljeqvist et al., 2013; Zeng et al., 2020).

One of the topics of interest is using mixed cultures in the bioleaching of sulfide minerals. It has been shown that using mixed cultures resulted in a higher metal extraction rate than using pure cultures (Akcil et al., 2007; Liu et al., 2011; Panda et al., 2015). On mesophilic and/or moderate thermophilic conditions, *Acidithiobacillus spp.*, *Leptospirillum* spp., *Sulfobacillus* spp., and *Ferroplasma* spp. were frequently detected in mixed cultures (Panda et al., 2013; Donati et al., 2016; Ma et al., 2018). These microorganisms played different roles (e.g., iron or sulfur oxidation, utilization of inorganic or organic substances) in a leaching system (Latorre et al., 2016). Meanwhile, they interacted with each other. For instance, *L. ferriphilum* enhanced the adhesion of *Sb. thermosulfidooxidans* to pyrite (Li et al., 2020). On the contrary, bioleaching using mixed cultures at low temperatures was scarcely studied. Liljeqvist et al. (2013) used a T7 mixed culture and a pure culture of *At. ferrivorans* strain SS3 in chalcopyrite bioleaching at 6°C. Bacterial community analysis revealed that *At. ferrivorans* dominated the T7 mixed culture (Dopson et al., 2007). Halinen et al. (2009) found that during bioleaching of a low-grade, multi-metal black schist at 7°C, some *Acidithiobacillus spp.* were present in the initial stage; after 500 days, *At. ferrooxidans*, *Ferromicrobium acidophilum*-like species and a gram-positive bacterium were detected.

Bioleaching of sulfide minerals is an electrochemical process in essence, which oxidized metal sulfide to Fe(III), sulfur and sulfate as well as other inorganic sulfur compounds, involving the transfer of electrons. Due to the difficulties in determining the intermediate products, the chemical processes of bioleaching are often hard to describe. Electrochemical approaches can convert the change of chemical substances to electrochemical signals, which can be captured by specific electrochemical equipments. Therefore, electrochemical methods have been commonly applied to investigate the dissolution processes of sulfide minerals. Arena et al. (2016) used electrochemical impedance spectroscopy (EIS) to study chalcopyrite leaching, and found that the addition of bacteria reduced the charge transfer reaction resistance. Zhao et al. (2015) compared the cyclic voltammograms (CV) of chalcopyrite and bornite electrodes. It was found that the anodic and cathodic peaks of the two mineral electrodes appeared in close positions, suggesting that similar oxidation and reduction reactions occurred during the dissolution of these two types of minerals.

Low-temperature bioleaching is critical in the recovery of valuable metals from sulfide minerals in alpine regions. Using microbial consortia instead of pure cultures can be a feasible approach to promote the leaching rate at low temperatures. To this end, chalcopyrite bioleaching by a mixed culture was performed in the present work. CV measurement, potentiodynamic polarization and EIS were used to characterize the dissolution processes and electrochemical behaviors of chalcopyrite. The study would provide insights into the chalcopyrite bioleaching mechanisms by mixed cultures at low temperatures.

## Materials and Methods

### Mineral

Chalcopyrite sample was obtained from Guangzhou in Guangdong province of China. XRD analysis showed that the sample contained 97.83% chalcopyrite and 2.17% quartz. The sample consisted of 33.1% copper, 28.7% iron, and 35.4% sulfur. Sample was ground and sieved to obtain fractional sizes ≤75 μm and sterilized by UV irradiation for 24 h in an aseptic room.

### Cultures

The microbial consortium used in the present study was enriched from the acid mine drainage of Yulong copper mine. Water samples were collected from five different sites in Yulong copper mine (an acidic mine drainage pond, an acidic tailing pool, two tailing dumps, and an effusion pool). All samples were combined and then cultivated in iron-free 9K medium (Peng et al., 2019) using chalcopyrite (at a pulp density of 3%) as a sole energy source at 6°C. After 4 months, apparent bacterial growth was observed and this was designated as the mixed culture. The obtained mixed culture was subcultured every 2 months and used as a seed in our lab for chalcopyrite bioleaching at low temperatures. *At. ferrivorans* strain YL15 was isolated from the acid mine drainage pond (Peng et al., 2017). The strain was routinely cultivated in iron-free 9K medium with chalcopyrite as a sole energy source at 6°C.

### Bioleaching Experiments

Bioleaching of chalcopyrite was conducted in 500 mL shake flasks. Chalcopyrite at a pulp density of 3% and iron-free 9K medium (250 mL) were added to each flask. The mixed culture or the pure culture of the strain YL15 was centrifuged at 10,000 × *g* for 10 min at 4°C to harvest cells. The collected cells were washed using aseptic acidified water (pH2.0) for twice and then resuspended with 10 mL 9K medium. Bioleaching using the cells of the mixed culture (designated as group MHJ) and the strain YL15 was, respectively, performed. The initial cell density was approximately 2 × 10^7^ cells/mL. The flasks were operated at 160 rpm and 6°C. An abiotic control was also conducted. Thymol in ethanol (2%, v/v) was added to the abiotic control to inhibit bacterial contamination. All the bioleaching experiments were carried out in triplicate.

Samples were withdrawn from flasks at regular intervals to determine the oxidation reduction potential (ORP), pH, cell density and metal ions concentrations. Every time 1.5 ml of the leaching solution was withdrawn to a sterile tube. The solution was centrifuged at 2,000 × g to let the ore residue settle down. The supernatant was withdrawn for the determination of various parameters. An equal volume of iron-free 9K medium was added to the tube, resuspend the ore residue, and returned to the leaching solution. Concentrations of iron and copper were measured using the method as described previously (Peng et al., 2019). Cells were counted using a blood cell counting chamber under a CX31 optical microscope (Olympus, Tokyo, Japan). The pH value was measured with pHS-3E acid meter (LEICI, Shanghai, China) and ORP (Ag/AgCl) value was assayed against a platinum electrode. XRD analysis of ore residue was performed according to a previous study (Peng et al., 2019).

### Sequencing of Prokaryotic 16S rRNA Gene Sequences

Bioleaching samples of the group MHJ were withdrawn at different time points. Sessile cells were detached from the mineral surface by vigorous vortex for 10 min in the presence of 1 gram of glass beads. The obtained samples contained both free and detached cells and were centrifuged at 2,500 × *g* for 5 min to remove the ore residues. The obtained supernatants were centrifuged at 10,000 × *g*, 4°C for 10 min to collect the total cells. Genomic DNA was extracted as previously described (Wu et al., 2019). The integrity of DNA was checked on an agarose gel by ethidium bromide staining. The concentration of DNA was measured using a ND-1000 spectrophotometer (NanoDrop Technologies, Wilmington, DE, United States).

Detailed procedures for amplification and sequencing of prokaryotic V4 hypervariable region of 16S rRNA gene have been described in a previous document (Xiao et al., 2015). Briefly, sequences of the V4 region of the prokaryotic 16S rRNA gene were amplified using the universal primers 515F and 806R (Rinke et al., 2014) linked with barcodes, adapter, a pad and a linker of two bases. The purified PCR products were employed for library construction. The MiSeq 500 kit was used for sequencing (2 × 250 bp paired-ends) on the MiSeq sequencing platform (Illumina, San Diego, CA, United States).

Raw sequences were split into samples based on their barcodes. The raw sequencing data were deposited at Sequence Read Archive under the accession number SRP133342. After sequence trimming, reads assembly and chimeras sequences checking, operational taxonomic unit (OTU) clustering was performed using UPARSE at a 97% similarity level. After that, the taxonomic affiliation of each sequence was analyzed using RDP Classifier against the SILVA 16S rRNA database at a 70% threshold (Leng et al., 2016).

### Electrochemical Experiments

Chalcopyrite electrode was prepared by cutting the chalcopyrite sample into an approximately cubic shape. All the electrochemical experiments were performed using 50 mL iron-free 9K medium as the electrolyte and a three-electrode system in the absence of bacteria or in the presence of the mixed culture or the pure culture of strain YL15. The three-electrode system contained a chalcopyrite electrode (working electrode, effective working area ∼1 cm^2^), a graphite electrode (counter electrode), and a Ag/AgCl electrode (reference electrode). The chalcopyrite electrode was embedded in a special electrode sleeve and polished using 3,000 mesh emery cloth before the tests. Three uniform chalcopyrite electrodes were, respectively, immersed in the leaching solutions of different groups and then withdrawn at day 20 for the electrochemical tests. Electrochemical measurements were performed at 6°C using a VersaSTAT platform equipped with Model273A potentiostat/galvanostat (EG&G Princeton Applied Research, Oak Ridge, TN, United States) controlled by the Power-Suite program. CV tests were performed at a scan rate of 20 mV/s. The scan range of CV was -800 to +800 mV (vs. open circuit potential, OCP). For potentiodynamic polarization tests, the electrode potential varied from -250 to +600 mV (vs. OCP) at a sweep rate of 2 mV/s. EIS tests were carried out in a frequency range of 10^–1^–10^–5^ Hz. EIS data were fitted using the Zview software (version 2.0). All potential values in the present work were against the Ag/AgCl reference electrode.

## Results

### Bioleaching Experiments

**FIGURE 1:**
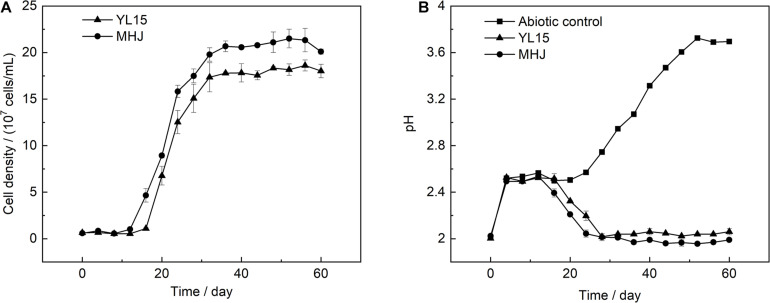
Variations of **(A)** cell density and **(B)** pH during chalcopyrite bioleaching at 6°C. MHJ, bioleaching by the mixed culture. H_2_SO_4_ (9.2 M) was added to the leaching solution to maintain the pH at ∼2.5 from day 4 to 16 for the bioleaching systems, and from day 4 to 20 for the abiotic control. For each bioleaching system, a total of 418 μl of 9.2 M H_2_SO_4_ per 250 ml culture was added. For the abiotic control, a total of 455 μl of 9.2 M H_2_SO_4_ per 250 ml culture was added.

The mixed culture (group MHJ) and the pure culture of *At. ferrivorans* strain YL15 obtained in our lab were used for bioleaching of chalcopyrite at 6°C. The bacterial growth during bioleaching is shown in Figure 1A. The bacterial cells of the group MHJ entered the logarithmic phase on day 12, and on day 16 for YL15. The highest cell densities of MHJ and YL15 were 2.2 × 10^8^ and 1.9 × 10^8^ cells/mL, respectively. The variation of pH during bioleaching is shown in Figure 1B. During the early phase, 9.2 M H_2_SO_4_ was added to the leaching solution to maintain the pH at ∼2.5. The pH value in the group MHJ began to decrease on day 12 and achieved ∼2.0 on day 24. The trend of pH during bioleaching by the strain YL15 was similar to the group MHJ, but the process was postponed, and the value was higher than in the group MHJ (decreased to ∼2.1).

**FIGURE 2:**
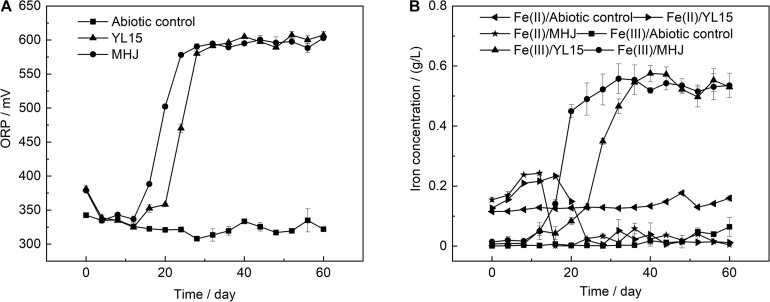
Variations of **(A)** ORP and **(B)** ferric and ferrous iron concentrations during chalcopyrite bioleaching.

**FIGURE 3:**
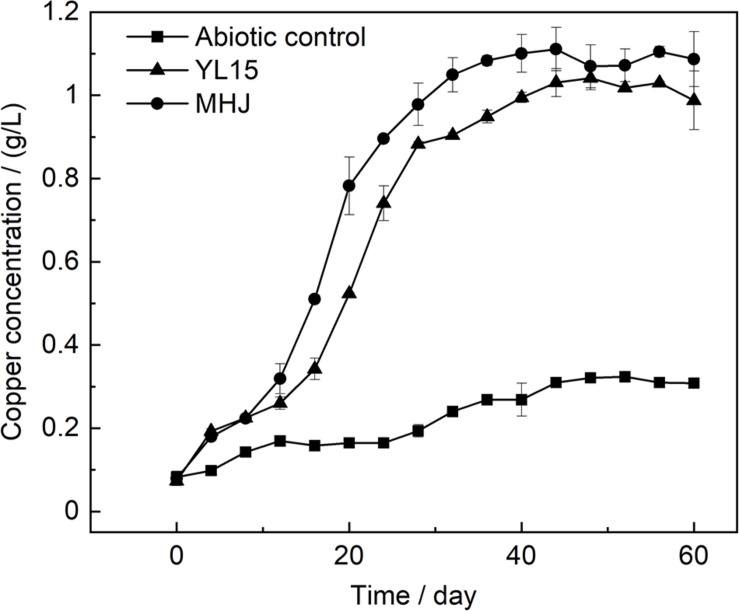
Variation of copper concentration during chalcopyrite bioleaching.

The variation of the ORP value is shown in Figure 2A. The ORP value was low in the early stage, but after that, it rocketed up to ∼600 mV in both groups. The value of ORP is mainly controlled by the ratio of Fe(III) to Fe(II). Variations of Fe(III) and Fe(II) concentrations are shown in Figure 2B. The ratio of Fe(III) to Fe(II) was low in the early stage. After that, as microbial oxidation of ferrous iron accelerated, the concentration of Fe(III) increased, accompanied by a decrease in the concentration of Fe(II). This was in accordance with the low ORP value in the early stage and its rapid rise after that (Figure 2A). The ore residues were analyzed using XRD. The results showed that all the ore residues contained only chalcopyrite and quartz, and no iron precipitate such as jarosite was detected. Variation of copper concentration is shown in Figure 3. The copper concentration in the presence of bacteria was higher than that in the abiotic control (Figure 3), suggesting that microorganisms greatly enhanced the dissolution of chalcopyrite. The copper concentration increased significantly after day 12 and reached the highest value on day 44 (11.2%) in MHJ and on day 48 (10.3%) in the YL15-mediated leaching system, respectively. Recently, bioleaching of another chalcopyrite sample using the strain YL15 at 6°C was performed, and the highest copper leaching rate achieved was 19.3% (on day 80) (Zeng et al., 2020). It suggested that the difference in the mineral sample might result in significant contrast in the highest leaching rate and the leaching course. The results of the present study suggested that the mixed culture was more efficient in chalcopyrite bioleaching than the pure culture. Previously, a T7 mixed culture was used to leach a chalcopyrite concentrate, and 16% of the copper was recovered in a 62-day course (Liljeqvist et al., 2013). However, the T7 mixed culture had comparable leaching performance to the *At. ferrivorans* strain SS3. This might be due to that the T7 mixed culture was dominated by *At. ferrivorans*, while the relative abundance of other species was very low (Dopson et al., 2007).

### Microbial Community Dynamics of the Group MHJ

The microbial community of the group MHJ was monitored using high-throughput sequencing of the 16S rRNA gene. Samples were withdrawn on days 20, 30, and 50 (designated as MHJ1, MHJ2, and MHJ3). A total of 673,782 pair-end reads were obtained. Rarefaction curves of all samples based on sequencing reads and number of OTUs reached plateaus, indicating that the sequencing depth was appropriate for estimating the microbial diversity.

**FIGURE 4:**
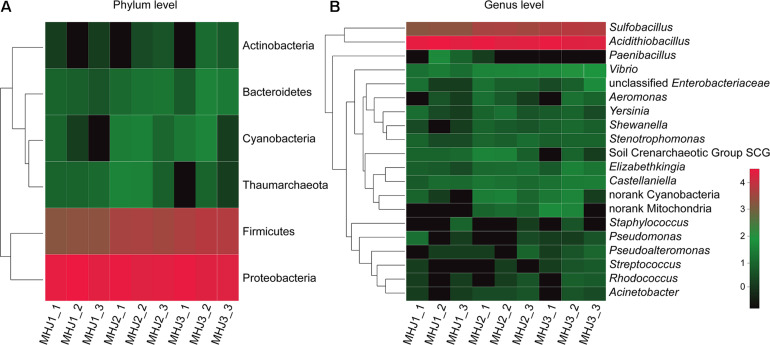
Heatmap of microbial communities of MHJ at **(A)** phylum and **(B)** genus levels. MHJ1_1, MHJ1_2 and MHJ1_3, MHJ2_1, MHJ2_2 and MHJ2_3, MHJ3_1, MHJ3_2 and MHJ3_3 were the triplicates of samples MHJ1, MHJ2, and MHJ3, respectively.

At the phylum level, Proteobacteria was the dominant lineage, which accounted for 81.5–94.9% of the total biomass, followed by Firmicutes (5.04–18.39%). Other phyla were also detected, e.g., Cyanobacteria, Thaumarchaeota and Bacteroidetes, but they were at extremely low level (<1%) (Figure 4A). Down to the genus level, a total of 27, 35, and 31 OTUs were detected for MHJ1, MHJ2, and MHJ3, respectively, and 20 OTUs were shared by all samples. *Acidithiobacillus* dominated the microbial communities, accounted for 94.8, 87.0, and 81.1% in MHJ1, MHJ2, and MHJ3, respectively. Sequences of *Sulfobacillus* (affiliated to Firmicutes) were detected at a considerable level and increased from 5.04% on day 20 to ∼10% on day 50.

To find out the affiliations of these two lineages, the 16S rRNA gene of sample MHJ3 was amplified using primers 27F and 1492R. The products were transformed to *E. coli* DH5α to build a clone library as described in a previous document (Liu et al., 2010). Five clones were randomly selected, and only 2 OTUs were detected, one of which was 100% identical to the 16S rRNA gene sequence of strain YL15 (accession number: KY352776.2) and the other one had a 98% identity to an undescribed *Sulfobacillus sp*. The results implied that the *Acidithiobacillus* spp. should be *At. ferrivorans.* The content of *Acidithiobacillus* decreased and the relative abundance of *Sulfobacillus* increased as bioleaching continued. This may be due to that the accumulation of organic substances was beneficial to the growth of *Sulfobacillus* (Johnson et al., 2008) or that the gram-positive *Sulfobacillus* might be more resistant to the adverse environment in the late phase than the gram-negative *At. ferrivorans* (e.g., accumulation of heavy metal ions). *Sulfobacillus* is commonly considered as a moderate thermophile, although some species can grow at temperatures <20°C (Melamud et al., 2003). Furthermore, it was documented that a mixed culture was enriched at 5°C with ferrous sulfate as a sole energy and sequences close to *Sb. montserratensis* were detected at an abundance >10% (Escobar et al., 2010). These results indicated that there might be novel *Sulfobacillus* species existing in the low-temperature bioleaching culture.

There were also other genera in the mixed culture, for instance, *Stenotrophomonas* spp., *Pseudomonas* spp. and *Castellanilla* spp., but they were of extremely low abundance (<0.5%) (Figure 4B). These species have been identified in either bioleaching systems or other acidic metal-containing environments. For instance, *Castellanilla* was the dominant lineage in an ethanol-stimulated acidic nitrate- and uranium-contaminated aquifer (Spain et al., 2007). In the low-grade copper sulfide bioleaching systems containing acid-processed rice straw (ARW), *Stenotrophomonas* accounted for >50% of the microbial community (Yin et al., 2019). *Pseudomonas* was detected in the bioleaching residues from a column bioreactor (Hao et al., 2016). These microorganisms can utilize organic substances as the energy source, and species like *Pseudomonas* could also secret organic acids such as citric acid, oxalic acid and succinic acid to enhance the dissolution of sulfide minerals (Hao et al., 2016).

Previous work has emphasized the importance of interactions between physiologically distinct acidophilic microorganisms (e.g., autotrophs and heterotrophs) in enhancing sulfide minerals dissolution (Okibe and Johnson, 2004). The existence of heterotrophs (such as *Sulfobacillus*, *Stenotrophomonas*, and *Pseudomonas*) could alleviate the inhibition of organic substances to the autotrophic *At. ferrivorans* and maintained the robustness of the microbial community (Johnson, 2008). In addition, *Sulfobacillus* can use inorganic sulfur compounds as a sole energy. Sulfur oxidation activity is essential in a bioleaching system. Firstly, it offers a low-pH condition for the dissolution of sulfide minerals. Secondly, elemental sulfur formed during the leaching process and precipitated on the mineral surface and had passivating effects on chalcopyrite bioleaching (Liljeqvist et al., 2013). Sulfur oxidation activity of the mixed culture was tested at an initial pH of 2.5. The sulfate concentration was measured using the barium sulfate turbidimetry (Liu et al., 2013). It was shown that the mixed culture had a higher sulfur-oxidation activity than the pure culture of strain YL15 (Supplementary Figure 1). Therefore, *Sulfobacillus* might help to reduce the passivating effect of sulfur during chalcopyrite leaching.

### Electrochemical Tests

#### CV Studies

**FIGURE 5:**
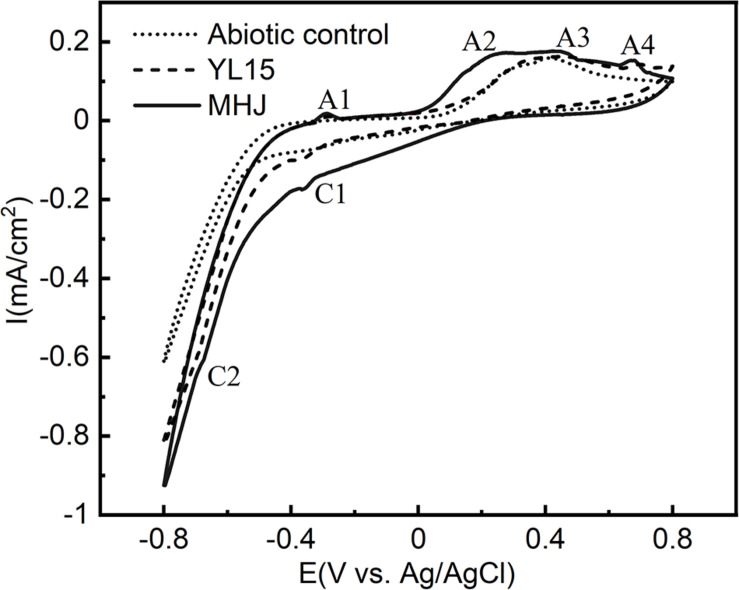
Cyclic voltammograms of chalcopyrite electrodes in different systems.

The cyclic voltammetry curves of chalcopyrite at 6°C are shown in Figure 5. The cyclic voltammetry curves of the three groups were similar. There were four anodic peaks (A1, A2, A3, and A4) and two cathodic peaks (C1 and C2). Peak A3 was a common anodic peak of chalcopyrite electrochemical behavior, which was mentioned as the “leading peak.” As shown in Eqs. 1, 2, there was a selective release of iron from the chalcopyrite crystal lattice and formation of intermediate nonstoichiometric chalcopyrite [Cu_1–*x*_Fe_1–*y*_S_2–*z*_ (y > x)] and covellite (Gómez et al., 1996; Zeng et al., 2011; Gu et al., 2013).

(1) $$\begin{array}{c} {\text{CuFeS}}_{2}\to {\text{Cu}}_{1-x}{\text{Fe}}_{1-y}{\text{S}}_{2-Z}+{\text{xCu}}^{2+}+{\text{yFe}}^{2+}+{\text{zs}}^{0}\\ +2\left(\text{x}+\text{y}\right){\text{e}}^{-} \end{array}$$

(2) $${\text{CuFeS}}_{2}\to 0.75\mathrm{CuS}+1.25S^{0}+0.25{\mathrm{Cu}}^{2+}+{\text{Fe}}^{2+}+2.5e^{-}$$

At more positive potential, peak A4 was a typical oxidation peak of chalcopyrite dissolution. It could be attributed to the oxidation of ferrous iron (López-Juárez et al., 2006) (Eq. 3).

(3) $${\text{CuFeS}}_{2}\to {\text{Cu}}^{2+}+{\text{Fe}}^{3+}+2S^{0}+5e^{-}$$

In the reverse potential scanning, there was a peak C1 at ∼−370 mV. It has been documented that this peak was involved in the reduction of covellite and chalcopyrite (Biegler and Horne, 1985; Arce and González, 2002) (Eqs. 4, 5):

(4) $$2CuS+2H^{+}+2e^{-}\to {\text{Cu}}_{2}\text{S}+{\text{H}}_{2}\text{S}$$

(5) $$2CuFeS_{2}+6H^{+}+2e^{-}\to {\text{Cu}}_{2}\text{S}+2Fe^{2+}+3H_{2}\text{S}$$

Peak C2 occurred at potential <−600 mV. According to Velásquez et al. (2001), this peak could be attributed to the reduction of chalcocite (Eq. 6):

(6) $${\text{Cu}}_{2}\text{S}+{\text{H}}_{2}\text{O}+2e^{-}\to 2Cu+{\text{HS}}^{-}+{\text{OH}}^{-}$$

During the later reverse scanning, a weak peak A1 occurred. The peak was not frequently reported during the dissolution of chalcopyrite. It might be responsible for the oxidation of copper (Eq. 7) or chalcocite (Zeng et al., 2013) (Eq. 8).

(7) $$2Cu+{\text{HS}}^{-}+{\text{OH}}^{-}\to {\text{Cu}}_{2}\text{S}+{\text{H}}_{2}\text{O}+2e^{-}$$

(8) $${\text{Cu}}_{2}\text{S}\to {\text{Cu}}_{1.92}\text{S}+0.08{\mathrm{Cu}}^{2+}+0.16e^{-}$$

At more positive potential, there was a Peak A2 at 270–320 mV. It was reported that this peak might be involved in the oxidation of Cu_*x*_S (1 < x < 2) species such as djurleite and geerite (Arce and González, 2002) (Eqs. 9, 10), which are the intermediate products of many copper sulfides.

(9) $${\text{Cu}}_{1.92}\text{S}\to {\text{Cu}}_{1.6}\text{S}+0.32{\mathrm{Cu}}^{2+}+0.64e^{-}$$

(10) $${\text{Cu}}_{1.6}\text{S}\to \text{CuS}+0.60{\mathrm{Cu}}^{2+}+1.20e^{-}$$

It can be seen from Figure 5 that the dissolution process of chalcopyrite with or without bacteria or in the presence of pure or mixed culture were similar. However, in the presence of bacteria, the current intensities of all the anodic and cathodic peaks were higher than the abiotic control, especially for the peaks A4, C1, and C2. It indicated that the addition of bacteria is beneficial to the dissolution of chalcopyrite. Moreover, the intensities of the anodic peaks A2, A3, and A4, and cathodic peaks C1 and C2 in the presence of the mixed culture were higher than those in the presence of pure culture. The enhancement of these peaks implied that the mixed culture increased the dissolution reaction rates of chalcopyrite compared with the pure culture of strain YL15. A further comparison revealed peak shifts on the CV spectra. The peak A4 of the abiotic control was located at ∼715 mV, but shifted to ∼700 mV of the pure-culture group, and ∼675 mV of the mixed-culture group; The peak A2 shifted from ∼315 mV (YL15) to ∼275 mV (MHJ); The peak A3 of the pure-culture group occurred at ∼445 mV, and showed a shift to ∼420 mV of the mixed-culture group. These demonstrated that the dissolution reactions occurred more easily in the presence of bacteria, especially the mixed culture.

#### Potentiodynamic Polarization Studies

**FIGURE 6:**
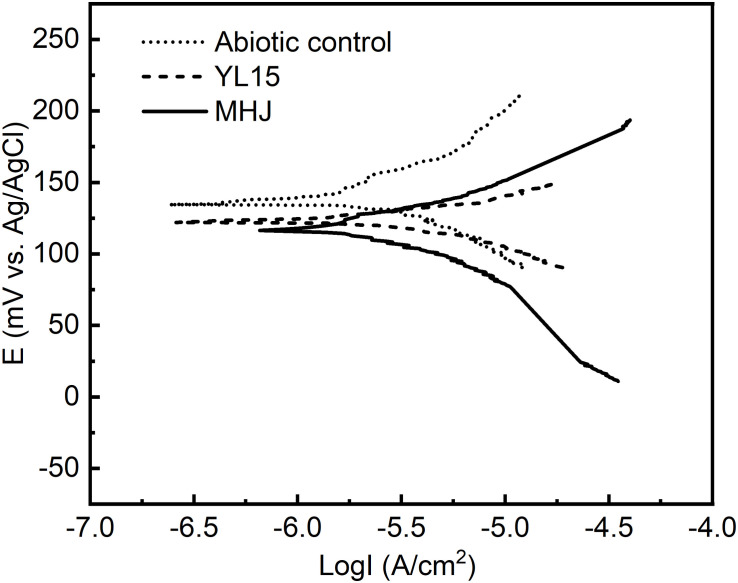
Tafel curves of chalcopyrite electrodes in different systems.

Results of potentiodynamic polarization tests in the absence of bacteria or in the presence of *At. ferrivorans* YL15/mixed culture at 6°C are shown in Figure 6 and Table 1. While the I_corr_ values of groups inoculated with bacteria increased compared with the abiotic control, their E_corr_ values decreased. The results suggested that the corrosion rates increased after adding bacteria. This was consistent with the results of leaching rates in different groups (Figure 3), and it further demonstrated that bacteria could enhance the dissolution of chalcopyrite. The mixed culture had advantages over the pure culture of strain YL15. The E_corr_ value of the group inoculated with the strain YL15 was 121.4 mV, but decreased to 116.3 mV of the group inoculated with the mixed culture. The I_corr_ value increased from 1.49 μA/cm^2^ of the pure-culture group to 1.62 μA/cm^2^ of the mixed-culture group. This implied that the mixed culture promoted the transfer of electrons, thus accelerated the corrosion of chalcopyrite.

**TABLE 1:** Tafel parameters of chalcopyrite electrodes in different systems.

	E_corr_/mV	I_corr_/(μA/cm^2^)
Abiotic control	134.8	1.31
YL15	121.4	1.49
MHJ	116.3	1.62

#### EIS Studies

**FIGURE 7:**
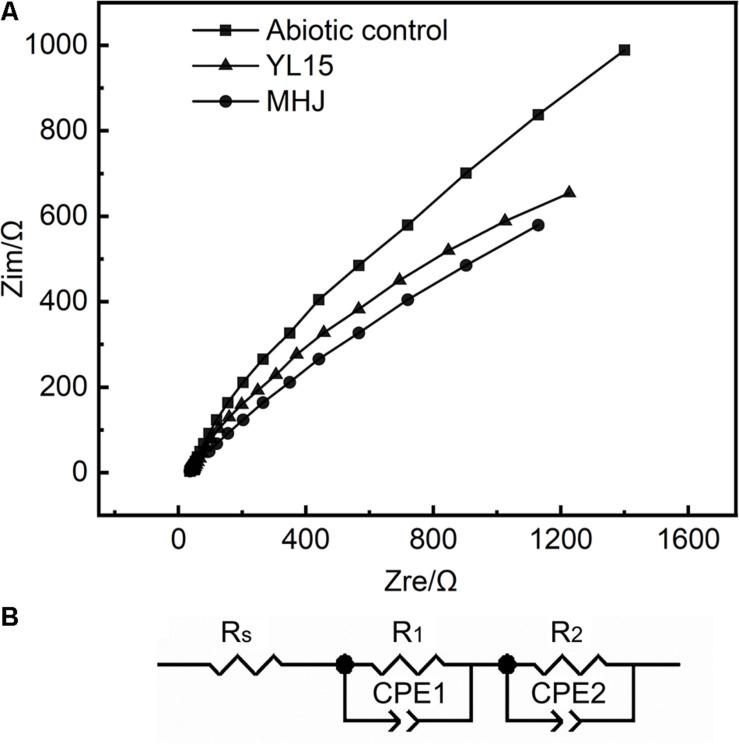
Results of EIS. **(A)** EIS curves of chalcopyrite electrodes in different systems. **(B)** Simulated circuit R_S_(R_1_CPE1) (R_2_CPE2).

The results of EIS in different systems are shown in Figure 7. The shapes of different EIS spectra were similar, so the addition of bacteria did not change the control steps of chalcopyrite dissolution. Each group showed an arc in the high-frequency region. This arc should be assigned to the resistance encountered during charge transfer. There was another arc in the low-frequency region, and this arc could be attributed to the impedance caused by passivation. The EIS data were fitted to an analog circuit with a linear combination of resistors and capacitors. The equivalent circuit of the impedance data in the present study should be R_S_(R_1_CPE1) (R_2_CPE2), where R_S_ was the solution resistance, R_1_ was the charge exchange impedance, and R_2_ represented the impedance derived from the passivation layer. The constant phase elements CPE1 and CPE2 were connected with the capacitance of the electrode interface (Zeng et al., 2020).

The detailed information on the fitting results using the analog circuit R_S_(R_1_CPE1) (R_2_CPE2) is shown in Table 2. The total impedance of different tests was MHJ < YL15 < abiotic control, which suggested that microorganisms decreased the impedance during chalcopyrite dissolution. Among the three types of impedances, R_1_ accounted for more than 50% of the total resistance, followed by R_2_ (took up 14–34% of the total resistance). This implied that the chalcopyrite dissolution process was mainly controlled by the charge transfer.

**TABLE 2:** Fitting results using the analog circuit R_S_(R_1_CPE1) (R_2_CPE2) of the chalcopyrite electrodes.

Sample	R_S_ (Ω)	R_1_ (Ω)	CPE1-T	CPE1-P	R_2_ (Ω)	CPE2-T	CPE2-P
Abiotic control	52.91	606.6	3.9625E–5	1.415 E–2	106.26	2.937E–4	6.310E–9
YL15	52.12	486.3	1.133E–4	1.625E–4	167.6	1.8298E–5	1.003E–6
MHJ	54.49	227.4	2.349E–7	2.362E–6	147.49	5.898E–5	5.270E–8

As shown in Table 2, the solution resistance in different tests was constant, whereas the charge exchange resistance and passivation layer impedance varied a lot among different groups. The charge exchange resistance of the abiotic control was 606.6 Ω, but decreased to 486.3 Ω in the presence of the strain YL15, and 227.4 Ω in the presence of the mixed culture. The passivation layer impedance exhibited a different trend. The impedance of the abiotic control was lower than the other two groups. This was due to that chalcopyrite in the abiotic control was least leached, and relatively low content of passivation substance was produced. Compared with the group inoculated with strain YL15, the *R*_2_ value of the group MHJ decreased, indicating less passivation on the mineral surface. S^0^ passivation during chalcopyrite bioleaching at low temperature has been shown (Liljeqvist et al., 2013). The mixed culture could promote the sulfur-oxidation activity (Supplementary Figure 1), and thus it might help reduce the passivation caused by elemental sulfur. Although iron precipitates such as jarosite may not passivate the mineral surface (Peng et al., 2019), the attenuation of the passivation in the group MHJ might also be due to the continued dissolution of copper sulfide intermediates. Taken together, the results of EIS demonstrated that the addition of bacteria, especially the mixed culture, could decrease the impedance of the leaching system and thus accelerated the dissolution of chalcopyrite.

## Conclusion

A mixed culture was used in chalcopyrite bioleaching at low temperature. The mixed culture was dominated by *Acidithiobacillus* spp. and *Sulfobacillus* spp., and it showed a greater leaching efficiency than the pure culture of the *At. ferrivorans* strain YL15. Electrochemical studies revealed that the mixed culture enhanced the dissolution reactions, decreased the corrosion potential while increasing the corrosion current, and reduced the impedance of the chalcopyrite electrode compared with the pure culture. This study provided insights into the mechanisms through which the mixed culture enhanced the chalcopyrite bioleaching.

## Data Availability Statement

The datasets presented in this study can be found in online repositories. The names of the repository/repositories and accession number(s) can be found below: https://www.ncbi.nlm.nih.gov/, SRP133342.

## Author Contributions

TP, WZ, and XW contributed conception and design of the work. TP, WL, JW, JM, and YP did the experiments. TP and WL drew the illustrations. TP, WL, XW, and WZ wrote the first draft of the manuscript. GQ and GG revised the manuscript. All authors read and approved the submitted version.

## Conflict of Interest

The authors declare that the research was conducted in the absence of any commercial or financial relationships that could be construed as a potential conflict of interest.

## References

[B1] Acosta M. Galleguillos P. Ghorbani Y. Tapia P. Contador Y. Velásquez A. (2014). Variation in microbial community from predominantly mesophilic to thermotolerant and moderately thermophilic species in an industrial copper heap bioleaching operation. *Hydrometallurgy* 150 281–289. 10.1016/j.hydromet.2014.09.010

[B2] Ahonen L. Tuovinen O. H. (1991). Temperature effects on bacterial leaching of sulfide minerals in shake flask experiments. *Appl. Environ. Microbiol.* 57 138–145. 16348389 10.1128/aem.57.1.138-145.1991 PMC182674

[B3] Ahonen L. Tuovinen O. H. (1992). Bacterial oxidation of sulfide minerals in column leaching experiments at suboptimal temperatures. *Appl. Environ. Microbiol.* 58 600–606. 16348648 10.1128/aem.58.2.600-606.1992 PMC195290

[B4] Ai C. Yan Z. Chai H. Gu T. Wang J. Chai L. (2019). Increased chalcopyrite bioleaching capabilities of extremely thermoacidophilic *Metallosphaera sedula* inocula by mixotrophic propagation. *J. Ind. Microbiol. Biotechnol.* 46 1113–1127. 10.1007/s10295-019-02193-3 31165968

[B5] Akcil A. Ciftci H. Deveci H. (2007). Role and contribution of pure and mixed cultures of mesophiles in bioleaching of a pyritic chalcopyrite concentrate. *Miner. Eng.* 20 310–318. 10.1016/j.mineng.2006.10.016

[B6] Arce E. M. González I. (2002). A comparative study of electrochemical behavior of chalcopyrite, chalcocite and bornite in sulfuric acid solution. *Int. J. Miner. Process.* 67 17–28. 10.1016/S0301-7516(02)00003-0

[B7] Arena F. A. Suegama P. H. Bevilaqua D. dos Santos A. L. A. Fugivara C. S. Benedetti A. V. (2016). Simulating the main stages of chalcopyrite leaching and bioleaching in ferrous ions solution: an electrochemical impedance study with a modified carbon paste electrode. *Miner. Eng.* 92 229–241. 10.1016/j.mineng.2016.03.025

[B8] Banerjee I. Burrell B. Reed C. West A. C. Banta S. (2017). Metals and minerals as a biotechnology feedstock: engineering biomining microbiology for bioenergy applications. *Curr. Opin. Biotechnol.* 45 144–155. 10.1016/j.copbio.2017.03.009 28371651

[B9] Biegler T. Horne M. D. (1985). The electrochemistry of surface oxidation of chalcopyrite. *J. Electrochem. Soc.* 132 1363–1369. 10.1149/1.2114117

[B10] Donati E. R. Castro C. Urbieta M. S. (2016). Thermophilic microorganisms in biomining. *World J. Microbiol. Biotechnol.* 32:179. 10.1007/s11274-016-2140-2 27628339

[B11] Dopson M. Halinen A. K. Rahunen N. Ozkaya B. Sahinkaya E. Kaksonen A. H. (2007). Mineral and iron oxidation at low temperatures by pure and mixed cultures of acidophilic microorganisms. *Biotechnol. Bioeng.* 97 1205–1215. 10.1002/bit.21312 17187443

[B12] Elberling B. Schippers A. Sand W. (2000). Bacterial and chemical oxidation of pyritic mine tailings at low temperatures. *J. Contam. Hydrol.* 41 225–238. 10.1016/S0169-7722(99)00085-6

[B13] Escobar B. Buccicardi S. Morales G. Wiertz J. (2010). Biooxidation of ferrous iron and sulphide at low temperatures: implications on acid mine drainage and bioleaching of sulphide minerals. *Hydrometallurgy* 104 454–458. 10.1016/j.hydromet.2010.03.027

[B14] Gómez C. Figueroa M. Muñoz J. Blázquez M. L. Ballester A. (1996). Electrochemistry of chalcopyrite. *Hydrometallurgy* 43 331–344. 10.1016/0304-386X(96)00010-2

[B15] Gu G. Hu K. Zhang X. Xiong X. Yang H. (2013). The stepwise dissolution of chalcopyrite bioleached by *Leptospirillum ferriphilum*. *Electrochim. Acta* 103 50–57. 10.1016/j.electacta.2013.04.051

[B16] Halinen A. K. Rahunen N. Kaksonen A. H. Puhakka J. A. (2009). Heap bioleaching of a complex sulfide ore: Part II. Effect of temperature on base metal extraction and bacterial compositions. *Hydrometallurgy* 98 101–107. 10.1016/j.hydromet.2009.04.004

[B17] Hallberg K. B. Gonzalez-Toril E. Johnson D. B. (2010). *Acidithiobacillus ferrivorans*, sp. nov.; facultatively anaerobic, psychrotolerant iron-, and sulfur-oxidizing acidophiles isolated from metal mine-impacted environments. *Extremophiles* 14 9–19. 10.1007/s00792-009-0282-y 19787416

[B18] Hao X. Liang Y. Yin H. Ma L. Xiao Y. Liu Y. (2016). The effect of potential heap construction methods on column bioleaching of copper flotation tailings containing high levels of fines by mixed cultures. *Miner. Eng.* 98 279–285. 10.1016/j.mineng.2016.07.015

[B19] Hedrich S. Guézennec A.-G. Charron M. Schippers A. Joulian C. (2016). Quantitative monitoring of microbial species during bioleaching of a copper concentrate. *Front. Microbiol.* 7:2044. 10.3389/fmicb.2016.02044 28066365 PMC5167697

[B20] Hu W. Feng S. Tong Y. Zhang H. Yang H. (2020). Adaptive defensive mechanism of bioleaching microorganisms under extremely environmental acid stress: advances and perspectives. *Biotechnol. Adv.* 42:107580. 10.1016/j.biotechadv.2020.107580 32590051

[B21] Johnson D. B. (2008). Biodiversity and interactions of acidophiles: key to understanding and optimizing microbial processing of ores and concentrates. *Trans. Nonferrous Met. Soc. China* 18 1367–1373. 10.1016/S1003-6326(09)60010-8

[B22] Johnson D. B. Joulian C. d’Hugues P. Hallberg K. B. (2008). *Sulfobacillus benefaciens* sp. nov., an acidophilic facultative anaerobic *Firmicute* isolated from mineral bioleaching operations. *Extremophiles* 12 789–798. 10.1007/s00792-008-0184-4 18719854

[B23] Kupka D. Liljeqvist M. Nurmi P. Puhakka J. A. Tuovinen O. H. Dopson M. (2009). Oxidation of elemental sulfur, tetrathionate and ferrous iron by the psychrotolerant *Acidithiobacillus* strain SS3. *Res. Microbiol.* 160 767–774. 10.1016/j.resmic.2009.08.022 19782750

[B24] Kupka D. Rzhepishevska O. I. Dopson M. Lindstrom E. B. Karnachuk O. V. Tuovinen O. H. (2007). Bacterial oxidation of ferrous iron at low temperatures. *Biotechnol. Bioeng.* 97 1470–1478. 10.1002/bit.21371 17304566

[B25] Langdahl B. R. Ingvorsen K. (1997). Temperature characteristics of bacterial iron solubilisation and ^14^C assimilation in naturally exposed sulfide ore material at Citronen Fjord, North Greenland (83°N). *FEMS Microbiol. Ecol.* 23 275–283. 10.1016/S0168-6496(97)00032-9

[B26] Latorre M. Cortés M. P. Travisany D. Di Genova A. Budinich M. Reyes-Jara A. (2016). The bioleaching potential of a bacterial consortium. *Bioresour. Technol.* 218 659–666. 10.1016/j.biortech.2016.07.012 27416516

[B27] Leng Y. Yi M. Fan J. Bai Y. Ge Q. Yao G. (2016). Effects of acute intra-abdominal hypertension on multiple intestinal barrier functions in rats. *Sci. Rep.* 6 22814. 10.1038/srep22814 26980423 PMC4793228

[B28] Li Q. Yang B. Zhu J. Jiang H. Li J. Zhang R. (2018). Comparative analysis of attachment to chalcopyrite of three mesophilic iron and/or sulfur-oxidizing acidophiles. *Minerals* 8 406–418. 10.3390/min8090406

[B29] Li Q. Zhu J. Li S. Zhang R. Xiao T. Sand W. (2020). Interactions between cells of *Sulfobacillus thermosulfidooxidans* and *Leptospirillum ferriphilum* during pyrite bioleaching. *Front. Microbiol.* 11:44. 10.3389/fmicb.2020.00044 32063894 PMC7000362

[B30] Liljeqvist M. Rzhepishevska O. I. Dopson M. (2013). Gene identification and substrate regulation provide insights into sulfur accumulation during bioleaching with the psychrotolerant acidophile *Acidithiobacillus ferrivorans*. *Appl. Environ. Microbiol.* 79 951–957. 10.1128/AEM.02989-12 23183980 PMC3568537

[B31] Liu H. Gu G. Xu Y. (2011). Surface properties of pyrite in the course of bioleaching by pure culture of *Acidithiobacillus ferrooxidans* and a mixed culture of *Acidithiobacillus ferrooxidans* and *Acidithiobacillus thiooxidans*. *Hydrometallurgy* 108 143–148. 10.1016/j.hydromet.2011.03.010

[B32] Liu H. Xia J. Nie Z. Peng A. Ma C. Zheng L. (2013). Comparative study of sulfur utilization and speciation transformation of two elemental sulfur species by thermoacidophilic archaea *Acidianus manzaensis* YN-25. *Process Biochem.* 48 1855–1860. 10.1016/j.procbio.2013.09.005

[B33] Liu X. Chen B. Wen J. Ruan R. (2010). *Leptospirillum* forms a minor portion of the population in Zijinshan commercial non-aeration copper bioleaching heap identified by 16S rRNA clone libraries and real-time PCR. *Hydrometallurgy* 104 399–403. 10.1016/j.hydromet.2010.03.024

[B34] López-Juárez A. Gutiérrez-Arenas N. Rivera-Santillán R. E. (2006). Electrochemical behavior of massive chalcopyrite bioleached electrodes in presence of silver at 35°C. *Hydrometallurgy* 83 63–68. 10.1016/j.hydromet.2006.03.039

[B35] Ma L. Wang X. Liu X. Wang S. Wang H. (2018). Intensified bioleaching of chalcopyrite by communities with enriched ferrous or sulfur oxidizers. *Bioresour. Technol.* 268 415–423. 10.1016/j.biortech.2018.08.019 30103167

[B36] Melamud V. S. Pivovarova T. A. Tourova T. P. Kolganova T. V. Osipov G. A. Lysenko A. M. (2003). *Sulfobacillus sibiricus* sp. nov., a new moderately thermophilic bacterium. *Microbiology* 72 605–612. 10.1023/A:1026007620113 14679908

[B37] Okibe N. Johnson D. B. (2004). Biooxidation of pyrite by defined mixed cultures of moderately thermophilic acidophiles in pH-controlled bioreactors: significance of microbial interactions. *Biotechnol. Bioeng.* 87 574–583. 10.1002/bit.20138 15352055

[B38] Panda S. Akcil A. Pradhan N. Deveci H. (2015). Current scenario of chalcopyrite bioleaching: a review on the recent advances to its heap-leach technology. *Bioresour. Technol.* 196 694–706. 26318845 10.1016/j.biortech.2015.08.064

[B39] Panda S. Parhi P. K. Nayak B. D. Pradhan N. Mohapatra U. B. Sukla L. B. (2013). Two step meso-acidophilic bioleaching of chalcopyrite containing ball mill spillage and removal of the surface passivation layer. *Bioresour. Technol.* 130 332–338. 10.1016/j.biortech.2012.12.071 23313677

[B40] Pathak A. Morrison L. Healy M. G. (2017). Catalytic potential of selected metal ions for bioleaching, and potential techno-economic and environmental issues: a critical review. *Bioresour. Technol.* 229 211–221. 10.1016/j.biortech.2017.01.001 28108075

[B41] Peng T. Chen L. Wang J. Miao J. Shen L. Yu R. (2019). Dissolution and passivation of chalcopyrite during bioleaching by *Acidithiobacillus ferrivorans* at low temperature. *Minerals* 9 332–341. 10.3390/min9060332

[B42] Peng T. Ma L. Feng X. Tao J. Nan M. Liu Y. (2017). Genomic and transcriptomic analyses reveal adaptation mechanisms of an *Acidithiobacillus ferrivorans* strain YL15 to alpine acid mine drainage. *Plos One* 12:e0178008. 10.1371/journal.pone.0178008 28542527 PMC5438186

[B43] Rinke C. Lee J. Nath N. Goudeau D. Thompson B. Poulton N. (2014). Obtaining genomes from uncultivated environmental microorganisms using FACS–based single-cell genomics. *Nat. Protoc.* 9:1038. 10.1038/nprot.2014.067 24722403

[B44] Spain A. M. Peacock A. D. Istok J. D. Elshahed M. S. Najar F. Z. Roe B. A. (2007). Identification and isolation of a *Castellaniella* species important during biostimulation of an acidic nitrate- and uranium-contaminated aquifer. *Appl. Environ. Microbiol.* 73 4892–4904. 10.1128/AEM.00331-07 17557842 PMC1951013

[B45] Srichandan H. Mohapatra R. K. Singh P. K. Mishra S. Parhi P. K. Naik K. (2020). Column bioleaching applications, process development, mechanism, parametric effect and modelling: a review. *J. Ind. Eng. Chem.* 90 1–16. 10.1016/j.jiec.2020.07.012

[B46] Velásquez P. Leinen D. Pascual J. Ramos-Barrado J. R. Cordova R. Gómez H. (2001). XPS, SEM, EDX and EIS study of an electrochemically modified electrode surface of natural chalcocite (Cu_2_S). *J. Electroanal. Chem.* 510 20–28. 10.1016/S0022-0728(01)00533-2

[B47] Wu X. Wu X. Shen L. Li J. Yu R. Liu Y. (2019). Whole genome sequencing and comparative genomics analyses of *Pandoraea* sp. XY-2, a new species capable of biodegrade tetracycline. *Front. Microbiol.* 10:33. 10.3389/fmicb.2019.00033 30761094 PMC6361800

[B48] Xiao Y. Xu Y. Dong W. Liang Y. Fan F. Zhang X. (2015). The complicated substrates enhance the microbial diversity and zinc leaching efficiency in sphalerite bioleaching system. *Appl. Microbiol. Biotechnol.* 99 10311–10322. 10.1007/s00253-015-6881-x 26266752

[B49] Yin S. Chen W. Chen X. Wang L. (2019). Bacterial-mediated recovery of copper from low-grade copper sulphide using acid-processed rice straw. *Bioresour. Technol.* 288:121605. 10.1016/j.biortech.2019.121605 31176935

[B50] Zeng W. Peng Y. Peng T. Nan M. Chen M. Qiu G. (2020). Electrochemical studies on dissolution and passivation behavior of low temperature bioleaching of chalcopyrite by *Acidithiobacillus ferrivorans* YL15. *Miner. Eng.* 155:106416. 10.1016/j.mineng.2020.106416

[B51] Zeng W. Qiu G. Chen M. (2013). Investigation of Cu–S intermediate species during electrochemical dissolution and bioleaching of chalcopyrite concentrate. *Hydrometallurgy* 13 158–165. 10.1016/j.hydromet.2013.02.009

[B52] Zeng W. Qiu G. Zhou H. Chen M. (2011). Electrochemical behaviour of massive chalcopyrite electrodes bioleached by moderately thermophilic microorganisms at 48°C. *Hydrometallurgy* 105 259–263. 10.1016/j.hydromet.2010.10.012

[B53] Zhao H. Hu M. Li Y. Zhu S. Qin W. Qiu G. (2015). Comparison of electrochemical dissolution of chalcopyrite and bornite in acid culture medium. *Trans. Nonferrous Met. Soc. China* 25 303–313. 10.1016/S1003-6326(15)63605-6

