# Supplementary figures and images for: Bioleaching and Electrochemical Behavior of Chalcopyrite by a Mixed Culture at Low Temperature
Source: Front Microbiol. 2021 May 10;12:663757. doi: 10.3389/fmicb.2021.663757 (PMC8141852; doi:10.3389/fmicb.2021.663757)

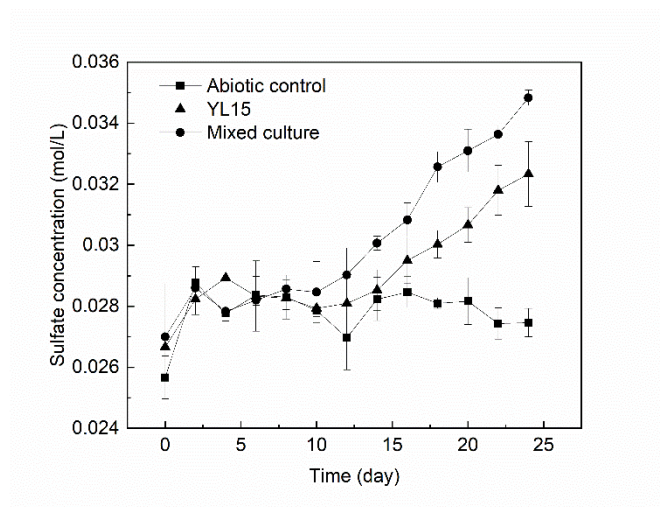

Figure S1 Variation of sulfate concentration during oxidation of  $S^0$ .

Supplement: Supplementary file 1 [file Image_1.pdf]
